# Gender Disparities and Outcomes Of Acute Coronary Syndromes In Brazil

**DOI:** 10.5935/abc.20180210

**Published:** 2018-11

**Authors:** Andrea De Lorenzo

**Affiliations:** 1 Instituto Nacional de Cardiologia, Rio de Janeiro, RJ - Brazil; 2 Universidade Federal do Rio de Janeiro, Rio de Janeiro, RJ - Brazil

**Keywords:** Acute Coronary Syndrome, Prognosis, Gender Identity, Myocardial Infarction, Risk Factors, Percutaneous Coronary Intervention, Aged, Women

Coronary artery disease (CAD) was considered, for years, a "male disease”, a concept that influenced diagnostic and clinical decision-making processes.^1^^,^^2^ However, currently there is consistent evidence showing that CAD is a leading cause of death in women. On the basis of pooled data from studies of the National Heart, Lung and Blood Institute (1995-2012), it is estimated that within one year after a first myocardial infarction, 18% of males and 23% of females will die, and the median survival time is, at ≥ 45 years of age, 8.2 years for males and 5.5 for females.^3^ The underestimation of cardiovascular risk among women frequently resulted in a more conservative treatment and contributed to poorer outcomes.^4^ In the last decade, several studies have assessed the issue of gender disparities in the diagnosis, treatment, and outcomes of acute coronary syndromes (ACS).^2^^,^^4^ In this context, the study by Soeiro et al.^5^ contributes to the understanding of this issue by presenting data from a Brazilian registry of ACS.

In this multicenter registry, the primary endpoint was in-hospital, all-cause mortality, and the secondary endpoint was the combination of cardiogenic shock, death, reinfarction, ischemic stroke and bleeding during a mean follow-up of 8 months. Just like any registry, it has limitations, such as the absence of data on other diseases like cancer, as well as on the differences in post-discharge management, adherence to treatment, among others, all which might influence survival in any group. Nonetheless, it has a large number of patients (2,437 men and 1,308 women) and may offer an interesting view of the Brazilian scenario of gender differences in ACS.

Of note, at presentation, women less often had ST-elevation and multivessel CAD than men, but were older and more frequently diabetic, dyslipidemic and hypertensive. These data are in line with other studies.^6^ Unfortunately, data on symptoms at presentation are not available. It is known that, in ACS, women are less likely to present with classical angina symptoms, which may lead to under and/or misdiagnoses in women, and in turn may explain the worse outcomes, particularly in younger women.^6^^,^^7^ Accordingly, in the present study, it was noteworthy that percutaneous coronary interventions and coronary artery bypass grafting were more frequently performed in men than in women.

Regarding outcomes, there were no significant differences between men and women. This contrasts to other studies in which women had worse outcome after ACS, what has been attributed, among other factors, to older age or the presence of more comorbidities in women.^4^^,^^8^ On the other hand, similar short-term outcomes in men and women have also been reported,^9^ especially after adjustment for clinical differences and the severity of angiographic disease.^10^ Gaui et al,^11^ in an analysis of Brazilian death certificates from 2004 to 2011, reported higher proportional mortality due to acute ischemic heart disease in women from the Northeastern region, aged 40-49 years, than in men, despite overall lower proportional mortality. Globally, this demonstrates that the outcomes of ACS in women are at least equivalent to those of men, if not worse.

The longstanding “knowledge gap” on CAD in women, both on the part of physicians and of patients, has created inequalities in healthcare access and processes. However, fortunately, our understanding of gender-specific differences in the initial presentation, pathophysiology, treatment effectiveness, and clinical outcomes have changed. The currently presented data are important to underscore the need to increase knowledge about the importance of CAD in women, so that possible gender biases may be effectively avoided, and better results obtained for the cardiovascular health of women.
